# Acknowledgement to Reviewers of *Biomedicines* in 2017

**DOI:** 10.3390/biomedicines6010010

**Published:** 2018-01-16

**Authors:** Biomedicines Editorial Office

**Affiliations:** MDPI AG, St. Alban-Anlage 66, 4052 Basel, Switzerland

Peer review is an essential part in the publication process, ensuring that *Biomedicines* maintains high quality standards for its published papers. In 2017, a total of 72 papers were published in the journal. Thanks to the cooperation of our reviewers, the median time to first decision was 18.5 days and the median time to publication was 47 days. The editors would like to express their sincere gratitude to the following reviewers for their time and dedication in 2017:
Abdel-Daim, Mohamed M.Mani, VigneshwaranAimanianda, Vishu KumarMarchal, SophieAlfarouk, KhalidMartini, MaurizioAltomonte, JenniferMena, IgnacioAlwahsh, SalamahMetyas, SamyAmpofo, EmmanuelMicheau, OlivierAn, SaiMichel, PiotrAngrand, Pierre-OlivierMilej, DanielAzab, AbdullatifMiljković, Miloš D.Babu, DineshMohankumar, KumaravelBaharom, FaezzahMongiat, MaurizioBarbe, Mary F.Moreno, MargaritaBartolotti, MassimoMuzio, MartaBasak, DebasishNawrot, Barbara C.Bathula, ChandraNierkens, StefanBecker, KathrinOliveira, Paula Alexandra Martins DeBenedec, DanielaOniga, IlioaraBenedetti, SaraOrcesi, SimonaBiondi, ElisaParashar, DeepakBose, DebojitPartanen, JukkaBranchini, AlessioPatil, BasavaprabhuCarmody, RuaidhriPenarrocha-Diago, MiguelChang, Nan-ShanPerez, LaurentChen, GuiqianPerkins, NeilChiappone, AnnalisaPetrovykh, Dmitri Y.Chou, JoshuaPetta, IoannaChoudhary, SanjeevPillow, ThomasChoudhury, MalayPollet, JeroenChristians, ElisabethPreissner, Klaus T.Chuburu, FrançoiseQuintana, Ainhoa LucíaClausen, JohannesRahib, LolaCollins, Maurice N.Rai, PrakashCourville, Elizabeth L.Rauwel, ProtimaDabrowiak, JamesRemaut, KatrienDe Lago, EvaReyes, FernandoDelville, Marie-HélèneRicci, SerafinoDini, LucianaRico-Villademoros, FernandoDonnelly, OliverRödel, FranzDubielecka, Patrycja M.Rundle, PaulDunavin, NeilSacco, PasqualeEibl, Martha M.Sampathi, ShilpaEljaafari, AssiaSarantopoulos, StefanieEritja, RamonSawynok, JanaFalzarano, Maria SofiaSbardella, GianlucaFast, Loren D.Serafini, GianlucaFerrone, SoldanoSerda, RitaFigueroa-Romero, ClaudiaShahbazi, Mohammad AliFischer, DagmarShanmugam, MuruganandanFiume, GiuseppeShi, FengFujita, TetsujiShigdar, SarahGanesan, LathaShiue, Yow-lingGarcía, TaniaShoenfeld, YehudaGiamperi, LauraSholkamy, EmanGill, TiffanyShoshan, MariaGordiichuk, PavloSommer, Wolfgang H.Greenwood, Michael T.Spingler, BernhardGrigoryan, Eleonora N.Sri Vedavyasa Sri, RadhakrishnanGuan, YuanSt. Croix, BradHamon, MorganStagos, DimitriosHanganu, DanielaStevens, MeganHarrington, CharlesSteyn, FrederikHarris, Randall E.Sturgill, Jamie L.Hassan, Sherif T. S.Sturiale, Carmelo LucioHellweg, Christine E.Subramaniam, PrasadHer, Lu-ShiunSun, WujinHernandez, MariaSzablewski, LeszekHolsinger, DamianTaniguchi, YasushiHopkins, SamanthaTanner, Julian A.Iacobazzi, VitoTatullo, MarcoIkegaya, NaokiTaylor, Cormac T.Ilmer, MatthiasTedesco, IdoloIm, AnnieTruong Phuoc, NghiaIonita, PetreUnderwood, Mark D.Islam, M. NurulVacchiano, GiuseppeIto, TaichiVähä-Koskela, MarkusJena, PrasantVallera, DanielKawahara, YukioVan Den Bergh, HubertKhandelwal, PoojaVandooren, JenniferKlokov, Dmitry Y.Vassilopoulos, GeorgeKohanbash, GaryVeeramani, SureshKomasa, SatoshiVidarsson, GesturKoneru, BhuvaneswariVirta, PasiKuang, HuihuiVladimirov, VladimirKumorek, MartaVolk, DavidLayer, Paul G.Walls, ZacLazarević, VladimirWang, JiLee, Joo HyoungWang, RanranLemay, GuyWarner, SusanneLentini, GiovanniWei, YufengLi, ChenyuXie, QiLi, FengXin, DongyueLiu, Cheuk LunYang, YongkangLiu, JiaYeo, SynLivermore, JoanneYue, PatrickLoganzo, FrankZafar, MuhammadLoskog, AngelicaZago, MiriamLu, YangZavattaro, ElisaLuczak, Michal W.Zavattaro, ElisaLutfy, KabirullahZhang, CaiguoLuznik, LeoZhang, XiangminMa, XingzheZhang, YixuanMacNeill, AmyZhou, Heshan SamMaggioni, DanielaZhu, LuchangMallipeddi, Prema LathaZhu, WeiMandracci, Pietro


